# Decoupling of Radial Growth Phenology From Temperature Constraints in the Clonal Shrub *Alnus alnobetula* at the Alpine Treeline

**DOI:** 10.1002/ece3.72198

**Published:** 2025-09-29

**Authors:** Walter Oberhuber, Gerhard Wieser, Andreas Gruber

**Affiliations:** ^1^ Department of Botany Universität Innsbruck Innsbruck Austria

**Keywords:** alpine treeline, bud burst, climate‐growth relationship, dendrometer, green alder, radial increment

## Abstract

Green alder ( *Alnus alnobetula* (Ehrh.) K. Koch) is a tall shrub that is widespread across the treeline ecotone in the Central European Alps. This shrub forms dense monospecific thickets and spreads rapidly through clonal propagation. Understanding its growth dynamics and climatic sensitivity is therefore essential for assessing its potential spread under changing environmental conditions. The study focused on determining the key dates of intra‐annual radial stem growth (RG) within the treeline ecotone on Mt. Patscherkofel (1940–2150 m a.s.l., Tyrol, Austria) and the influence of climate variables on daily RG. RG was continuously recorded during 2022–2024 by dendrometers and modeled using the Gompertz function to determine key phenological dates of intra‐annual RG, i.e., onset, end, duration, and time of maximum RG. Additionally, daily RG was extracted from dendrometer traces, and correlations (Spearman *ρ*) with environmental variables were performed. Bud burst was found to occur 28 ± 3 days before the onset of RG, indicating that carbon reserves are initially allocated toward leaf development. RG started and ceased at the end of June (doy [day of the year] 177 ± 7) and the end of August (doy 233 ± 9), respectively. The RG duration amounted to 56 ± 9 days and primarily occurred in July. Significant relationships (*p* < 0.001) found between temperature variables and daily RG at all study sites underscore the importance of temperature for RG. However, in all study years, the maximum RG (doy 192 ± 7) was already observed within *c*. 2 weeks of RG onset and prior to recorded temperature maxima. This study revealed that, despite daily RG of the clonal shrub *Alnus alnobetula* being temperature‐dependent, its RG phenology is decoupled from prevailing temperature and differs from that of trees. This is most likely due to its deciduous nature and the preferential carbon allocation for clonal propagation, which promotes horizontal spreading within the treeline ecotone.

## Graphical abstract

Green alder forms dense, monospecific thickets within the alpine treeline ecotone and spreads rapidly through clonal propagation. We determined key dates of intra‐annual radial stem growth (RG), and the influence of climate variables on daily RG. Despite its daily RG being temperature‐dependent, similar to treeline trees, our study revealed that the RG phenology of this shrub differs from that of upright trees. This is most likely due to the preferential carbon allocation for clonal propagation, which favors vigorous spreading after land abandonment or disturbance.

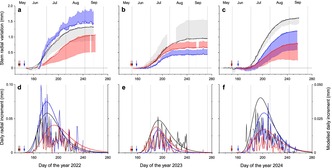

## 1 Introduction

Green alder ( *Alnus alnobetula* (Ehrh.) K. Koch, formerly known as *Alnus viridis* (Chaix) DC) is a multi‐stemmed tall shrub widespread in the Central European Alps (CEA) above the forestline (Boscutti et al. 2014). This tall shrub (canopy height between 2 and 3 m) forms dense monospecific thickets, and by clonal propagation (resprouting from root stock and adventitious shoots) *A. alnobetula* is able to quickly spread due to land abandonment and decrease in grazing pressure (Anthelme et al. 2007; Bühlmann et al. 2014; Caviezel et al. 2017; but see Pisetta et al. 2012). In addition, climate warming is contributing to an increase in shrub cover (Elmendorf et al. 2012; Macias‐Fauria et al. 2012; Ropars et al. 2015), because shrub growth in cold environments is limited by low temperatures (Forbes et al. 2010; Hallinger et al. 2010; Jørgensen et al. 2015; Garcia Criado et al. 2025). Within the alpine treeline ecotone in the CEA, the climate sensitivity of *A. alnobetula* was determined by applying dendroclimatological methods (response function analysis) and evaluation of climate in years showing exceptional growth deviation. In accordance with the growth limitation hypothesis (Körner 2003), a close direct relationship of radial stem growth (RG) of *A. alnobetula* to summer temperature was found (Oberhuber et al. 2022). Dendroclimatic analysis can identify the climatic factors most closely associated with variations in inter‐annual RG, i.e., tree‐ring width, but RG (cambial cell division and cell elongation) occurs on a time scale of hours to days and is controlled by environmental factors on a similar scale (Köcher et al. 2012; Rathgeber et al. 2016; Etzold et al. 2022). Intra‐annual RG can be determined using fine‐scaled histological analysis and high precision dendrometers, which provide a resolution of about 1 week and < 30 min, respectively. Due to the small diameter of *A. alnobetula* stems (mostly < 5 cm), the microcoring technique (Rossi, Anfodillo, and Menardi 2006; Deslauriers et al. 2017) required for histological analysis is not applicable. Dendrometers, however, provide an excellent means of monitoring the dynamics of intra‐annual RG, i.e., the secondary growth phenology, on a continuous basis (for a review see Deslauriers et al. 2007).

The period when new tissue is produced, e.g., when cambial activity and cell enlargement occur in the stem, is defined as the growing season *senso stricto* (Körner et al. 2023). The phenological season, defined by visible plant development such as bud burst and leaf shedding, can be very different from the actual growing season, and is therefore not a good proxy for RG (Richardson et al. 2010; Etzold et al. 2022). By recording intra‐annual RG with dendrometers, key phenological dates of RG, i.e., onset, end, duration, and time of maximum RG can be derived, and seasonal (short‐term) climatic influences on RG and RG phenology can be determined (Deslauriers et al. 2003; Michelot et al. 2012; Oberhuber et al. 2014). It has frequently been shown that temperature is the main driver of secondary growth at high elevation (e.g., Carrer and Urbinati 2004; Oberhuber 2004; Büntgen et al. 2005), which is in accordance with the growth‐limitation hypothesis of tree growth at the high‐elevation treeline (Körner 2021). RG phenology is also controlled by climatic conditions, but other factors, especially photoperiod, may be involved (Rossi, Deslauriers, et al. 2006; Huang et al. 2020; Mu et al. 2023).

Although previous studies (e.g., Rossi et al. 2007; Gruber, Baumgartner, et al. 2009; Cocozza et al. 2016) have extensively examined intra‐annual RG of treeline species like Swiss stone pine ( *Pinus cembra* ), Norway spruce ( *Picea abies* ), or European larch ( *Larix decidua* ), RG phenology of co‐occurring tall shrubs is scarce (Pasqualotto et al. 2021), and still completely lacking for *A. alnobetula* . Knowledge of RG phenology and drivers of intra‐annual RG in contrasting environments is needed to complete our understanding of *A. alnobetula* growth, and to better predict the potential spread of this native, light‐demanding shrub with a pioneer attitude to post disturbance colonization across the alpine treeline ecotone as a result of climate warming (Caviezel et al. 2017). To accomplish this, intra‐annual RG of *A. alnobetula* was monitored during 2022–2024 across the treeline ecotone on Mt. Patscherkofel (1940–2150 m a.s.l.; CEA) using high‐precision dendrometers. The objectives were to compare (i) the key phenological dates of intra‐annual RG within the treeline ecotone, and (ii) to determine the influence of environmental factors on daily stem radius increment. We expected that significant differences in growth phenology exist in the selected contrasting environments, and that temperature controls RG and RG phenology of this tall deciduous shrub within the treeline ecotone.

## 2 Material and Methods

### 2.1 Study Area

**FIGURE 1:**
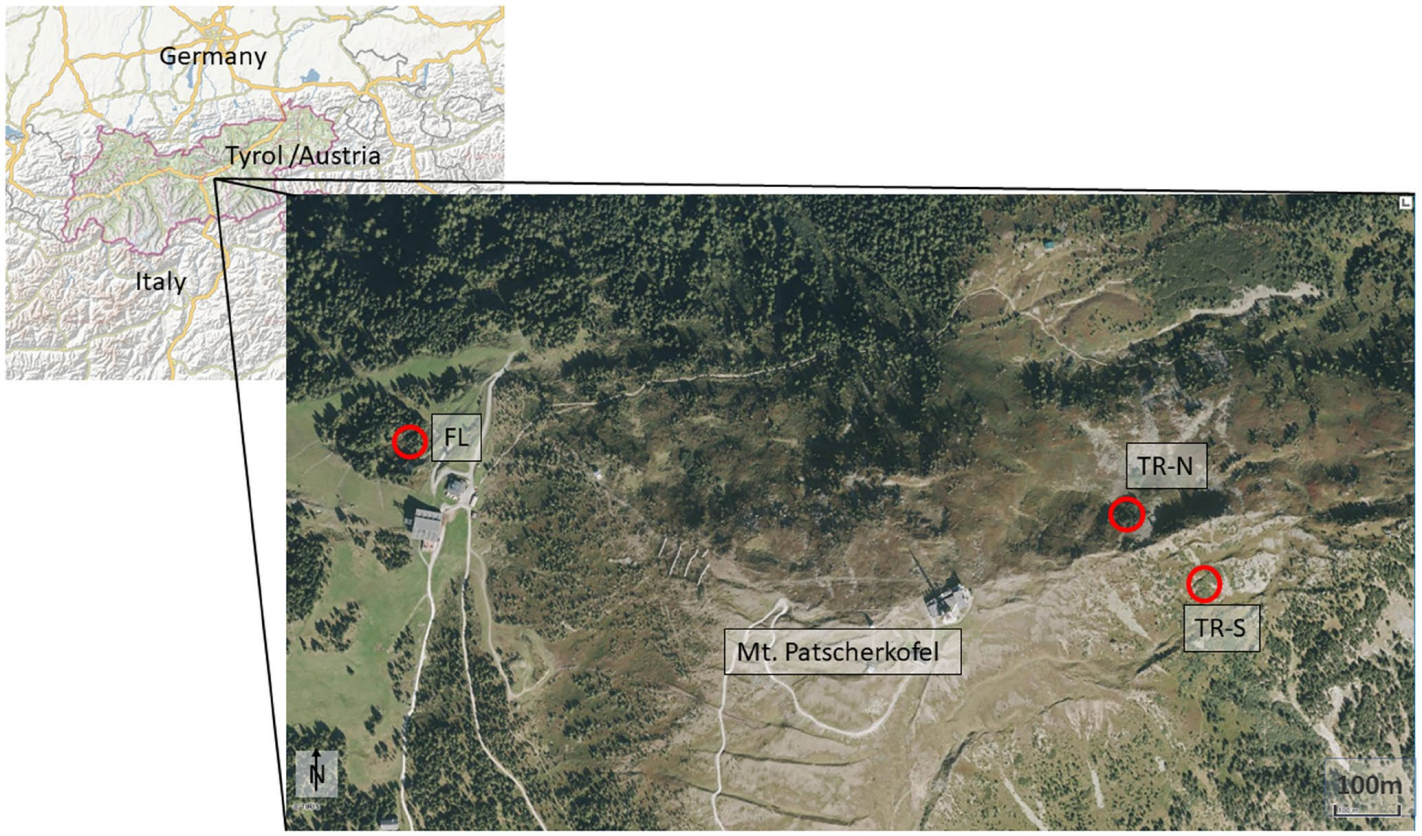
Spatial distribution of the study sites on Mt. Patscherkofel (FL = site close to the forestline, TR‐*N* = north‐facing treeline site, TR‐S = south‐east‐facing treeline site). *Source:* TirisMaps Land Tirol (https://maps.tirol.gv.at/synserver?user=guest&project=tmap_master; accessed on 20 August 2025).

The study area is situated at the treeline ecotone, extending from 1940 to 2150 m a.s.l. on Mt. Patscherkofel (2246 m a.s.l.; Tyrol, Austria; 47°12′31″ N, 11°27′38″ E; Figure 1), which is part of the CEA. The geology of Mt. Patscherkofel is characterized by the presence of gneisses and schists, while the soil type is classified as haplic podzol (Neuwinger 1970; Tollmann 1977; FAO 1998). In the period spanning from 1967 to 2021, mean annual precipitation at the top of Mt. Patscherkofel (where the meteorological station is located) amounted to 891 ± 123 mm. The maximum precipitation was recorded during summer months (June–August: 366 ± 69 mm), while the minimum was observed during winter (December–February: 143 ± 53 mm). The mean annual temperature during the same period was 0.3°C ± 0.8°C, with February (−6.9°C ± 2.6°C) and July (8.3°C ± 1.6°C) being the coldest and warmest months, respectively. The mean snow depth on Mt. Patscherkofel is generally less than 1 m. However, there is a marked spatial variation within the study area due to the irregular distribution caused by the occurrence of strong southerly winds (Föhn; Fliri 1975). In contrast to the south‐facing slopes, which may show minimal snow coverage in mid‐April, north‐facing slopes and avalanche gullies often retain substantial snowfields that extend up to May.

*Alnus alnobetula* stands are developed across the treeline ecotone primarily in leeward avalanche gullies exhibiting high water availability, but also occur at wind‐exposed sites on south‐ to southeast‐facing slopes. This tall clonal shrub is characterized by the formation of dense monospecific thickets, with a canopy height of 2–3 m. Across the treeline ecotone on Mt. Patscherkofel, which is dominated by Swiss stone pine ( *Pinus cembra* L.), three *A*. *alnobetula* stands were selected (Figure 1 and Table 1). These included a site situated somewhat below the forestline at 1940 m a.s.l. (FL) and two other sites located close to the treeline. The latter differed in terms of contrasting environmental conditions with regard to the duration of snow cover, i.e., one site facing southeast (TR‐S, 2140 m a.s.l.) and one facing north (TR‐N, 2150 m a.s.l.). The age of selected *A. alnobetula* stands within the treeline ecotone ranges from 20 to 30 years (Oberhuber et al. 2022).

**TABLE 1:** Site description and characteristics of selected *Alnus alnobetula* stands within the treeline ecotone.

Plot	Elevation (m a.s.l.)	Aspect	Slope (°)	Soil depth^a^ (cm)	Canopy height (m)	SDM^b^ (cm)	n DMR (2022/2023/2024)
FL	1940	NNW	35	15–20	3.0–3.5	4.1 ± 0.6	4/4/3
TR‐S	2140	SE	25	5–10	2.0–2.5	3.3 ± 0.2	9/8/8
TR‐N	2150	N	30	10–15	2.5–3.0	3.3 ± 0.4	3/8/7

Abbreviations: a.s.l. = above sea level, FL = forestline, n DMR = number of dendrometer records during the study period 2022–2024, SDM = stem diameter of shoots with dendrometers: Mean values ± standard deviation, TR‐N = treeline North, TR‐S = treeline South‐east.

^a^ Due to factors such as the presence of boulders or rock outcrops, the depth of the soil varies considerably within the study plots at small spatial scales. We have listed the primary range of soil depth.

^b^ Measured at the end of the study period in fall 2024.

### 2.2 Microclimate Records

During the study period, air temperature shielded against solar radiation and relative air humidity (RH) were collected at 30‐min intervals at 2 m height at all study sites across the treeline ecotone (CS215 temperature and RH sensor, Campbell Scientific Ltd., Shepshed, UK). Additionally, soil temperature (T107 temperature probe, Campbell Scientific Ltd., Shepshed, UK) and volumetric soil water content (SWC; ThetaProbes Type ML2 and ML3, Delta‐T, Cambridge, England) were recorded in the top 10 cm soil layer (four to five sensors for each variable and site). The daily precipitation totals were obtained from the meteorological station at the top of Mt. Patscherkofel, as these are considered representative for all study sites within the treeline ecotone (study sites were located within a radius of less than 1 km from the meteorological station). The calculation of vapor pressure deficit of the air (VPD) was performed based on the 30‐min intervals of air temperature and RH. The equation used was outlined by Prenger and Ling (2000). Mean daily air and soil temperature, SWC, RH, and VPD were calculated by averaging all measurements, i.e., 48 values day^−1^.

### 2.3 Replication Statement

**Table:** 

Scale of inference	Scale at which the factor of interest is applied	Number of replicates at the appropriate scale
Individual	Plot	Forestline 3–4 Treeline South 8–9 Treeline North 3–7

*Note:* Number of replicates varied during three study years (see Table 1).

### 2.4 Dendrometer Records

The present study involved the analysis of daily dendrometer records collected during three growing seasons (2022–2024). In order to carry out continuous monitoring of intra‐annual radial growth, temperature‐compensated electronic diameter dendrometers with a resolution of < 3 μm (DD‐S, Ecomatik, Munich, Germany) and point dendrometers (ZN12‐O‐WP, Natkon.ch, Switzerland) were mounted onto the outer bark of *A. alnobetula* shoots from different individuals at a distance of 15–20 cm from the rootstock. Due to the smooth and thin dead outer bark layer, which is hardly hygroscopic in this species and therefore has little impact on dendrometer records (e.g., Ilek et al. 2016; Oberhuber et al. 2020), we refrained from removing this tissue before mounting the dendrometers (periderm thickness was *c*. 1 mm) to avoid damaging the underlying living tissues, i.e., the conducting phloem cells and cambial tissue. The total number of dendrometer records analyzed at all three study sites across the treeline ecotone ranged from 16 to 20 per year (see Table 1).

The arching and ascending growth of shoots of *Alnus alnobetula* frequently leads to a pronounced asymmetric radial growth in the lower ascending stem region. According to Oberhuber et al. (2023), inter‐annual agreement in RG is related to growth rate, and therefore the mounting of dendrometers wherever possible was undertaken on the fastest growing radius, which mainly occurs on the lower side of the stem. Furthermore, although up to 30 shoots can sprout from one individual (i.e., stock), a high level of agreement was found among ring‐width time series of different shoots belonging to the same individual (Oberhuber et al. 2023). Therefore, mounting one dendrometer per individual can be considered an accurate record of its intra‐annual RG. The impact of solar radiation on the dendrometer traces is considered negligible, primarily due to the formation of a dense canopy by the sprouting of numerous shoots from a singular rootstock with canopy light transmittance < 15%. The data loggers (HOBO UX120‐006 M, ONSET, Bourne, MA, USA; CR1000X Campbell Scientific Ltd., Shepshed, UK) were programmed to record measurements at 30‐min intervals. The daily stem radius variation was calculated by averaging all daily measurements, i.e., 48 values day^−1^.

The daily stem radial increment was defined as the period during which the stem radius exceeded the morning maximum until the subsequent maximum was reached. In cases where multiple days were required to reach the previous cycle maximum, the difference between maximum values was divided by the number of days passed. Daily stem radius variations were modeled with a Gompertz function, employing the non‐linear regression procedure incorporated within the Origin software package (OriginPro 2024b, 10.1.5.132, OriginLab Corporation, Northampton, MA, USA). The Gompertz equation has been demonstrated to be an effective tool for the description of growth‐limiting processes (Zeide 1993; Rossi et al. 2003; De Micco et al. 2019). Utilizing the developed Gompertz models, the dynamics of daily RG throughout the 2022–2024 growing season were determined at all study sites. The start and cessation of RG were determined on the basis of the modeled growth, whereby the onset and end were defined as the points when 5% and 95% of total RG were reached, respectively. The duration of RG was thus determined by the difference in days between the day of year (doy) when onset and cessation occurred. The mean (Gr_mean_) and maximum (Gr_max_) radial growth rates (μm day^−1^), which represent the intrinsic growth potential, were determined on the basis of the Gompertz function. Gr_mean_ was calculated over the period when 5% and 95% of annual RG were achieved. Gr_max_ was determined on the day of the year when the inflection point in the model occurred. Bud burst was defined as the presence of 50% of the buds on an individual plant with visible emerging leaves.

The calculation of non‐parametric Spearman correlation coefficients (*ρ*) between environmental variables and extracted daily radial increments was necessitated by the absence of a normal distribution of the data, as evidenced by the application of the Shapiro–Wilk test. The analysis was performed over the period (i) when 5% and 95% of maximum RG had been achieved, and (ii) when RG was most linear during the study years, i.e., over a period of 14 days around the inflection point of the Gompertz modeled RG during 2022–2024. The latter approach is founded upon the recommendations put forward by Deslauriers et al. (2007), who proposed that the analysis should be constrained to the most linear growth phase during the study years, in order to accurately determine climate‐growth relationships. With regard to the precipitation variable, only days with a wet‐day threshold of 1 mm day^−1^ were taken into account in the correlation analysis. A time lag of 1 day was also considered in the climate‐growth analysis. The software package used for the analyses was Statistica, version 13.5.0.17 (TIBCO Software Inc., Tulsa, OK, USA). Throughout the manuscript, mean values and standard deviations (M ± SD) are provided.

## 3 Results

### 3.1 Environmental Variables During Study Years

**FIGURE 2:**
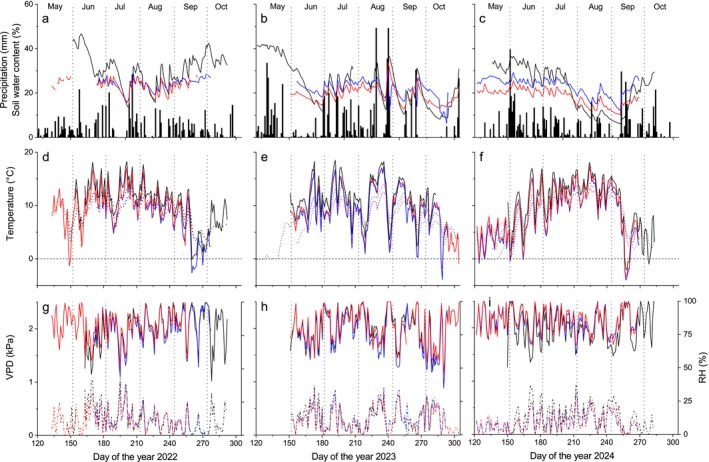
(a–i) Climate variables, soil water content and soil temperature recorded during the period 2022–2024 at study sites within the treeline ecotone. Mean daily soil water content and daily precipitation sum (a–c). Mean daily air temperature (solid lines) and soil temperature (dotted lines; d–f). Mean daily vapor pressure deficit (VPD; dotted lines) and relative air humidity (RH; solid lines; g–i). Study sites are denoted by black, red and blue lines for the forestline, and the south‐east and north‐facing treeline site, respectively.

Meteorological conditions at Mt. Patscherkofel varied considerably between the study years (Figure 2a–i). In 2022, relatively mild temperatures were observed in May: daily mean air temperatures were 5.3°C in 2022, 2.9°C in 2023, and 3.5°C in 2024 recorded at 2246 m a.s.l. on Mt. Patscherkofel (Table S1), with the exception of a late frost that occurred at the end of the month (cf. Figure 2d). In addition, the mean monthly air temperature of 10.5°C, recorded at the treeline in June 2022, was observed to be 1.1°C and 2.4°C higher than that recorded in 2023 and 2024, respectively (Figure 2d–f). The 2023 growing season was characterized by cool and wet conditions in May, which were followed by a dry period in June with a total rainfall of 40 mm (Table S1). From July through August, extreme temperature variability occurred, with several cold spells. At the treeline site facing north, the daily mean air temperature decreased to 2.6°C on 26 July, 1.8°C and 1.2°C on 7 and 29 August, respectively (Figure 2e). On the aforementioned days, the lowest recorded minimum temperature at this site was −1.2°C. In 2024, June was notable for its markedly cool temperature (monthly mean air temperature was 8.1°C at the treeline) and high precipitation (total rainfall 186 mm), which were followed by the warmest period from July through August during the study years (+1.8°C and +1.4°C compared to 2023 and 2022, respectively). Mean monthly soil temperatures were *c*. 0.7°C lower than the corresponding air temperatures, but with considerably less variability due to the attenuation of extremes (Figure 2d–f). During the summer months, SWC fluctuated mainly between 20 and 30 Vol. %, with the exception of August 2024, when it temporarily dropped to 10–15 Vol. % (Figure 2a–c). Throughout the study years, the daily mean RH across the treeline ecotone amounted to *c*. 80% during June–August. The daily mean VPD only exceeded a value of 1 kPa on sporadic days (Figure 2g–i).

### 3.2 Stem Radius Changes and Growth Phenology

**FIGURE 3:**
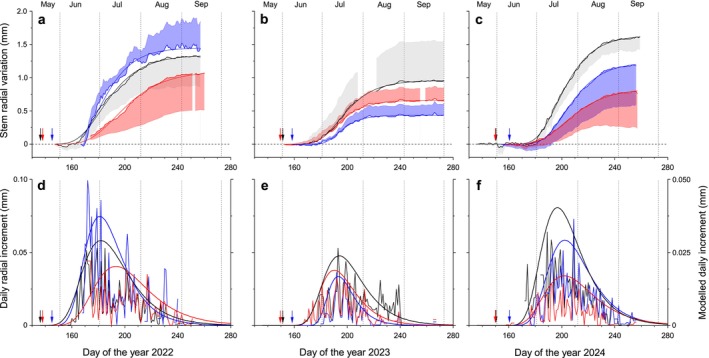
(a–f) Time series of stem radial variations (daily means±standard deviations) modeled by applying the Gompertz function (a–c). Extracted and modeled daily radial increment are depicted in (d–f). Arrows indicate time of bud burst at the study sites, which are denoted by black, red and blue lines/arrows for the forestline, and the south‐ and north‐facing treeline site, respectively.

**FIGURE 4:**
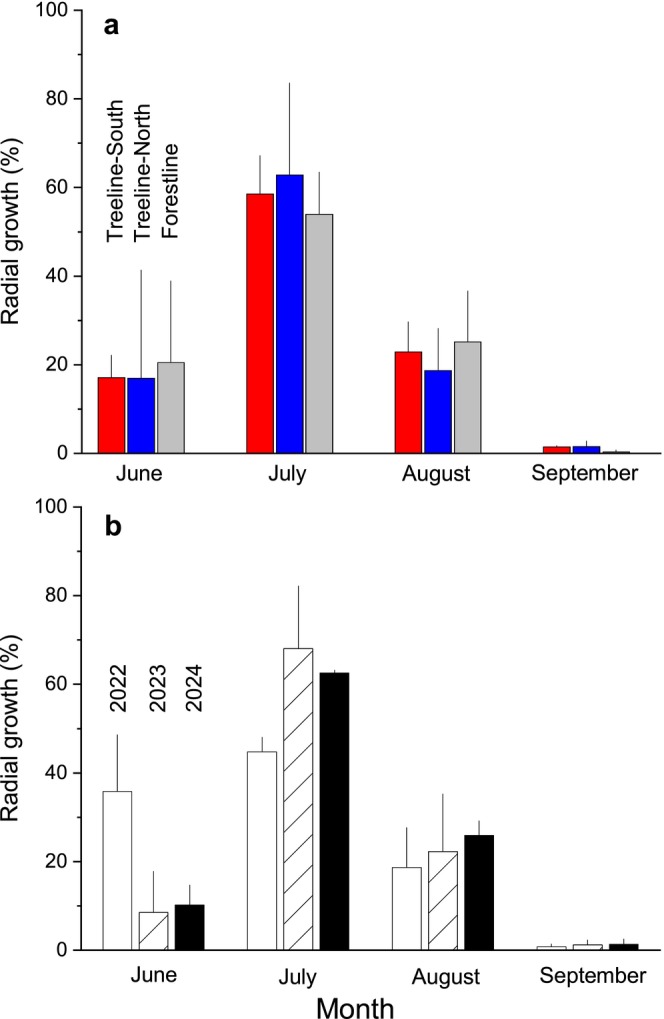
(a, b) Mean monthly radial stem growth within the treeline ecotone during all three study years (a), and mean radial stem growth during 2022–2024 at all study sites (b). Bars indicate standard deviations.

In Figure 3a–c, intra‐annual RG during 2022–2024 is depicted for study sites located in the treeline ecotone on Mt. Patscherkofel. The main growing period throughout the treeline ecotone is identified as July, with about 60% of annual growth occurring within this month (Figure 4a). The key dates of RG phenology at all study sites are summarized in Table 2. Within the treeline ecotone, the earliest occurrence of RG onset was recorded in mid‐June 2022 (doy 169 ± 4). The timing of RG onset in this year was significantly different (*p* < 0.05) from that in 2023 (doy 179 ± 4) and 2024 (doy 182 ± 2), leading to higher monthly growth in June 2022 compared to the following years (*c*. 35% and *c*. 10% in 2022 and 2023/2024, respectively; Figure 4b). Bud burst was found to occur 28 ± 3 days before the onset of RG within the treeline ecotone, considering all sites and study years (Figure 3a–f and Table S2). No significant difference in this lag was observed among years; however, a significant difference (*p* < 0.05) in this lag was observed between TR‐N (25 ± 1 days) and TR‐S (31 ± 3 days). Leaf shedding was observed to commence in early to mid‐October at the treeline and the forestline, respectively.

**TABLE 2:** Phenological dates of intra‐annual radial growth of *Alnus alnobetula* at study sites located within the treeline ecotone during 2022–2024. Timing of radial growth is based on the Gompertz function, i.e., 5% and 95% of maximum are regarded as onset and end of radial growth, respectively. Dates are given in days of the year (Dur = duration [days], FL = forestline, IP = inflection point; M ± SD = mean ± standard deviation, TR‐N = treeline North, TR‐S = treeline South‐east). Different letters indicate statistically significant differences between phenological parameters during the study period (Student's *t*‐test; *p* < 0.01).

Plot	2022				2023				2024			
	Onset	End	Dur	IP	Onset	End	Dur	IP	Onset	End	Dur	IP
FL	164	228	64	181	178	236	58	194	180	237	57	196
TR‐S	171	242	71	193	176	228	52	190	182	244	62	201
TR‐N	172	222	50	181	183	221	38	193	185	241	56	202
M ± SD	169 ± 4^a^	231 ± 10^a^	62 ± 11^a^	185 ± 7^a^	179 ± 4^b^	228 ± 7^a^	49 ± 10^a^	192 ± 2^a^	182 ± 3^b^	241 ± 4^a^	58 ± 3^a^	200 ± 3^a^

RG within the treeline ecotone ceased between doy 228 and doy 241 (i.e., mid‐ to late August), with the growing period lasting about 2 months (56 ± 9 days considering all study sites and years, Table 2). Mean (Gr_mean_) and maximum (Gr_max_) daily growth rates amounted to 16.3 and 25.9 μm day^−1^, respectively, and were not significantly different among sites (*p* > 0.05; data not shown).

The Gompertz function adequately fitted intra‐annual RG at all sites, with an *R* ^*2*^ range of 0.978 to 0.999 (Table 3). From dendrometer records extracted, Gompertz‐modeled daily radial increments are shown in Figure 3d–f. The inflection point of RG (IP) in the treeline ecotone across all sites and study years occurred at doy 192 ± 7 (*n* = 9; Table 2). The timing of the IP differed significantly (*p* < 0.05) within the treeline ecotone between 2022 (doy 185 ± 7) and 2024 (doy 200 ± 3), and between 2023 (doy 192 ± 2) and 2024 (Table 2). Daily mean air temperatures recorded at 7‐ and 14‐day intervals around the identified IP were found to be several degrees lower (−2.1°C to −4.7°C) than later during the growing season in all study years (Table S3).

**TABLE 3:** Parameters of the Gompertz function and *R* ^*2*^ of the model for intra‐annual radial growth of *Alnus alnobetula* within the treeline ecotone during 2022–2024 (see Figure 4a,d; A = upper asymptote, doy = day of the year, FL = forestline, I_p_ = inflection point, TR‐N = treeline North, TR‐S = treeline South‐east, κ = rate of change parameter). Mean values ± standard deviation are given.

Site	Year	*A* (μm)	*I*_p_ (doy)	κ	*R* ^*2*^
FL	2022	1342 ± 7	181 ± 0.2	0.059 ± 0.001	0.996
	2023	961 ± 3	194 ± 0.1	0.068 ± 0.001	0.998
	2024	1639 ± 6	196 ± 0.1	0.067 ± 0.001	0.999
TR‐S	2022	1100 ± 10	193 ± 0.3	0.050 ± 0.001	0.994
	2023	663 ± 3	190 ± 0.2	0.078 ± 0.001	0.997
	2024	841 ± 5	201 ± 0.2	0.055 ± 0.001	0.998
TR‐N	2022	1453 ± 13	181 ± 0.4	0.070 ± 0.003	0.978
	2023	435 ± 2	193 ± 0.2	0.105 ± 0.003	0.994
	2024	1239 ± 6	202 ± 0.2	0.064 ± 0.001	0.999

### 3.3 Relationship Between Daily Radial Increment and Environmental Factors

The relationships between stem radial increments extracted from dendrometer records and environmental variables are depicted in Table 4. In the period of maximum RG during 2022–2024, i.e., the linear growth phase around the IP of the Gompertz‐modeled growth, stem radial increments exhibited the strongest correlation with temperature variables at all study sites. However, when a lag of 1 day was considered in the correlation analysis, the coefficients increased markedly and became significant (*p* < 0.001) for VPD (direct relationship) and RH (inverse relationship) at all study sites, except for RH at TR‐N. Although the relationships between precipitation, SWC, and daily increments were found to be primarily non‐significant, significant inverse relationships were found between daily increments and precipitation at TR‐N (*ρ* = −0.417; *p* < 0.01), and daily increments and SWC at TR‐S (*ρ* = −0.247; *p* < 0.05; Table 4). The correlation coefficients consistently decreased when the period between 5% and 95% of the Gompertz‐modeled RG was considered (Table S4), except for RH at FL (*ρ* = 0.178, *p* < 0.05) and precipitation at TR‐S (*ρ* = 0.322, *p* < 0.01).

**TABLE 4:** Spearman correlation coefficients (*ρ*) between environmental variables (daily means with the exception of precipitation, where daily sums were used) and daily radial increments of *Alnus alnobetula* during study years 2022–2024. Correlations were calculated over a period of 14 days around the inflection point of the Gompertz modeled growth (see Table 3), and considering of a lag of 1 day. The corresponding coefficients are shown after the back slash. The number of data points is 87–91 for FL (except *n* = 39–40 for Prec), 92 for TR‐S (except *n* = 62 for *T*_camb_, and *n* = 37–39 for Prec), and 81–88 for TR‐N (except *n* = 62–64 for *T*_camb_, and *n* = 39 for Prec). FL = forestline, Prec = precipitation, RH = relative air humidity, SWC = soil water content, *T*_air_ = air temperature, *T*_camb_ = cambium temperature, TR‐N = treeline north, TR‐S = treeline south‐east, *T*_soil_ = soil temperature, VPD = daily mean vapor pressure deficit. ***p < 0.001; **p < 0.01; *p < 0.05.

	*T*_air_ (°C)	*T*_soil_ (°C)	*T*_camb_ (°C)	RH (%)	VPD (kPa)	Prec (mm)	SWC (%)
FL	0.110\0.572***	0.419***\0.434***	0.185\0.571***	0.126\−0.449***	−0.123\0.511***	0.263\−0.255	−0.132\−0.099
TR‐S	0.334**\0.680***	0.412***\0.340***	0.297*\0.659***	−0.013\−0.626***	0.081\0.662***	0.231\−0.187	−0.186\−0.247*
TR‐N	0.234*\0.409***	0.370***\0.343**	0.288*\0.414***	0.097\−0.115	0.090\0.393***	−0105\−0.417**	0.021\0.091

## 4 Discussion

After winter dormancy, bud burst marks the start of the new growing season. In this study, we found a marked delay between bud burst and the onset of RG in *A. alnobetula* (a diffuse porous species; Schoch et al. 2004) of *c*. 4 weeks within the treeline ecotone. In boreal *Betula* spp., the interval between bud burst and wood formation was found to be up to 3 weeks (Schmitt et al. 2004; Zhai et al. 2012), and Moser et al. (2010) reported that the needles of the deciduous European larch ( *Larix decidua* Mill.) appeared 3–4 weeks before the onset of the RG. The somewhat longer time span we found between bud burst and the RG onset within the treeline ecotone may be due to the determination of the onset of RG from dendrometer records, as the onset cannot be determined as precisely as from the records of xylem cell formation used in the cited studies. Consistent with our findings, D'Orangeville et al. (2021) reported that in several diffuse porous species, bud burst occurred on average 36 ± 6 days before the modeled threshold of 25% stem growth was reached. Bud burst also preceded the onset of RG by 4–6 weeks in *Corylus avellana* (L.) (hazelnut), a diffuse porous multi‐stemmed shrub (Pasqualotto et al. 2021). On the other hand, no significant delay between bud burst and the onset of RG was detected, e.g., in evergreen Swiss stone pine ( *Pinus cembra* L.) and Norway spruce ( *Picea abies* L.) at the treeline (Gruber, Baumgartner, et al. 2009; Rossi et al. 2009), and in deciduous beech ( *Fagus sylvatica* L.) and trembling aspen ( *Populus tremuloides* Michx.) (Cufar et al. 2008; Zhai et al. 2012). Hence, the timing of bud burst and the onset of RG vary widely between species, suggesting that carbon allocation strategies at the start of the growing season are species‐specific. The delay in RG onset in *A. alnobetula* with respect to bud burst is most likely attributable to the deciduous habit of this tall shrub, indicating that carbon reserves are initially allocated toward leaf formation, which is the major carbon sink in deciduous tree species (Klein et al. 2016), with cambium activity commencing subsequent to ongoing photosynthesis. However, asynchronous bud and wood phenology may also be related to organ‐specific differences in chilling requirements (Lin et al. 2024). Nevertheless, the delay in RG implies that the xylem of *A. alnobetula* formed in the previous growing season(s) is functionally active after the winter dormant period and can transport water to the developing canopy before the current xylem is functionally active. This assumption is supported by D'Orangeville et al. (2021), who report that the hydraulic conductivity in diffuse porous species is less affected by winter embolism.

In temperate and boreal trees, the timing of growth resumption after winter dormancy is well known to be driven by temperature (e.g., Rossi et al. 2007; Hänninen and Tanino 2011; Swidrak et al. 2011; Begum et al. 2013; Delpierre et al. 2019; Gao et al. 2022). Accordingly, earlier snowmelt and higher solar radiation, which increase soil temperatures, most likely explain the earlier bud burst and RG onset on TR‐S compared to TR‐N. Within the treeline ecotone, the occurrence of mild temperatures in May 2022 (mean daily air temperatures were 2.4°C and 1.8°C higher than in 2023 and 2024, respectively) also favored earlier bud burst and growth onset by 10 to 14 days compared to subsequent years. Earlier bud burst and onset of the growing season in association with warmer springs is a well‐documented phenomenon (e.g., Menzel et al. 2006; Montgomery et al. 2020). However, the significantly earlier onset of RG in 2022 did not result in significant differences in the culmination and end of RG between the study years. However, although RG culminated in July, approximately 3 weeks after the summer solstice, the consistent timing of peak growth across sites and study years does not rule out the potential influence of a photoperiodic constraint, as was reported for *Pinus cembra* within the study area (Gruber, Baumgartner, et al. 2009) and other tree species exposed to cold environments (e.g., Rossi, Deslauriers, et al. 2006; Heinrichs et al. 2007; Cuny et al. 2013). D'Orangeville et al. (2021) suggested that physiological differences in the resumption of cambium activity after winter dormancy are responsible for the delayed culmination of RG in diffuse porous species. Camarero and Rubio‐Cuadrado (2025) reported that while climate warming may lead to earlier bud burst and a longer phenological season, it will not necessarily result in more RG and, therefore, more CO_2_ sequestration. Similarly, several authors (Rathgeber et al. 2011; Ren et al. 2019; Dow et al. 2022; Tian et al. 2024; but see Chen et al. 2022) found that the rate at which growth occurs is more important than the duration of the growing period in reaching specific absolute growth. This is because plant growth is controlled by the activity of the carbon sinks, not by the carbon supplied (Körner 2015). It was also found that it is the rate of photosynthesis and not the length of the photosynthetic active period that determines terrestrial gross primary productivity (Xia et al. 2015).

At all study sites, *A. alnobetula* showed the highest Gr_max_ prior to the occurrence of recorded maxima of daily mean temperatures. These findings indicate that other factor(s) besides photoperiod and temperature, which are the most important cues influencing leaf senescence in winter deciduous species (Estiarte and Penuelas 2015), may control the cessation of RG in this clonal deciduous shrub. A combination of resource availability, hormonal signals, and environmental cues is suggested to be involved in the timing of RG cessation (Sorce et al. 2013; Cartenì et al. 2018). Yang et al. (2025) reported that shoot radial growth of *Abies forrestii* var. *smithii* commenced and ceased earlier than stem radial growth. Therefore, the authors concluded that shoot growth may also influence stem growth. Late bud burst and the delay between bud burst and RG onset suggest that the timing of maximum RG is influenced by the temporal dynamics of leaf and/or shoot growth. Despite the difference in elevation between the study sites (210 m), the consistency in the time of maximum RG was unexpected.

Results of this study also revealed that the main growing season of *A. alnobetula* lasts only 56 ± 9 days across the treeline ecotone, of which *c*. 60% occurs in July. The short duration of RG can be attributed to the significant carbon demand associated with lignification of the xylem cell wall (Cuny et al. 2014; Rathgeber et al. 2016)—a process that does not result in measurable stem expansion and therefore goes unnoticed in dendrometer‐based RG records. Furthermore, we suggest that the clonal propagation strategy of *A. alnobetula* , i.e., vegetative spread by root suckers and rooting of shoots, may trigger an early shift of carbon allocation to belowground organs. Co‐occurring *Pinus cembra* trees, which are unable to spread vegetatively, show a RG duration of 3–4 months, i.e., May through August within the treeline ecotone (Gruber, Baumgartner, et al. 2009; Gruber, Zimmermann, et al. 2009). Growth cessation in *A. alnobetula* occurred in mid‐August, but leaf shedding was observed to commence not before early October. Hence, the period of RG covers about 50% of the phenological season, i.e., bud burst to leaf shedding covers a time period of *c*. 4 months when photosynthesis is possible, suggesting that assimilated carbon may be allocated to processes such as cell wall lignification or clonal spreading. Furthermore, we found a large discrepancy in the total annual increment of *A. alnobetula* to co‐occurring evergreen Swiss stone pine ( *Pinus cembra* L.) at the treeline (Oberhuber et al. 2022 and data not shown). While mean annual increment of *A. alnobetula* shoots measured at TR‐S amounts to about 1 mm during study years 2022–2024, co‐occurring *Pinus cembra* trees (mean stem height: 3 ± 0.8 m; tree age at *c*. 20 cm stem height: 18 ± 4 years) show mean tree‐ring width of 2.6 ± 0.72 mm (*n* = 24 trees, 10‐year mean during 2012–2021). The striking difference not only in key phenological dates but also in RG among the life forms shrub and tree indicates that different carbon allocation strategies to aboveground and belowground exist. Dominance of fast horizontal spreading with shoots sprouting from the root system is characteristic of the clonal growth of *A. alnobetula* , where individual shoots seldom reach an age > 50 years. Single‐stemmed *Pinus cembra* trees, however, favor vertical growth, and vegetative propagation via rooting of branches does not occur in this species.

Climate‐growth relationships based on tree‐ring width revealed that inter‐annual RG of *A. alnobetula* is significantly and directly related to summer temperature (Oberhuber et al. 2022). In this study, we expected that *A. alnobetula* growing under varying site conditions at treeline would show divergent intra‐annual RG responses to environmental factors. However, we found that intra‐annual RG is significantly related to temperature within the treeline ecotone, indicating the importance of temperature for cambium activity under otherwise optimal conditions. During the formation of wood, growth is primarily related to the enlargement of the newly formed xylem cells (Gruber, Zimmermann, et al. 2009; Rathgeber et al. 2016) and is only marginally related to cambial cell division. Therefore, the direct relationship of VPD to radial stem increment can be explained by the enhancement of sap flow in *A. alnobetula* with increasing VPD (Körner et al. 1978; Van den Bergh et al. 2018, and data not shown), which increases cell turgor and hence cell enlargement and RG. A lag of 1 day appears to be necessary to improve the water status of the multi‐stemmed shrub and stimulate RG. In this regard, the lagged increase in the RG response to temperature and air humidity can be explained by their influence on VPD. The sporadic inverse relationships found between precipitation, SWC, and stem radial increment at treeline sites are most likely related to effects of soil moisture on soil temperature; i.e., moist cold soils are impairing water uptake and transport and hence sap flow (Aroca et al. 2012). The lack of significant (i) direct relationships between RG and SWC and precipitation, and (ii) inverse relationships with VPD and RG during the period around the inflection point of modeled RG; i.e., when RG was most linear, indicates that the water status of *A. alnobetula* at Gr_max_ is currently not restricted by climate change, as has been reported for tree growth (Grossiord et al. 2020; Trotsiuk et al. 2021; Schönbeck et al. 2022). However, the direct significant relationship found between precipitation and daily radial increment at TR‐S (*ρ* = 0.322, *p* < 0.01), when the growth period between 5% and 95% of the modeled RG is considered, indicates that water availability can already be constraining RG at sites characterized by gravelly and shallow soils.

## 5 Conclusions

This study provides insight into the effects of environmental variables on daily stem radius increment of *A. alnobetula* within the treeline ecotone. Although our findings underline the importance of temperature constraints on intra‐annual RG of *A. alnobetula* , similar to co‐occurring trees, key phenological dates of RG (onset, duration, peak RG) are decoupled from temperature constraints and strikingly differ from those of upright trees. We conclude that after land abandonment, the N_2_‐fixing clonal shrub *A. alnobetula* is able to spread vigorously within the treeline ecotone not least because of different preferences for carbon allocation to above‐ and belowground organs compared to upright trees. Therefore, future studies should quantify non‐structural carbohydrate (NSC) concentrations in different plant organs (leaves, stems, and roots) throughout the growing season (Smith et al. 2017; Wang et al. 2023; Landhäusser and Adams 2024) to gain a better understanding of the carbon allocation strategies of this clonally propagating deciduous shrub species.

## Author Contributions

**Walter Oberhuber:** conceptualization (equal), data curation (equal), formal analysis (equal), funding acquisition (lead), investigation (lead), methodology (equal), project administration (lead), validation (equal), visualization (lead), writing – original draft (lead). **Gerhard Wieser:** conceptualization (equal), data curation (supporting), formal analysis (supporting), funding acquisition (supporting), methodology (supporting), writing – review and editing (supporting). **Andreas Gruber:** conceptualization (equal), data curation (supporting), funding acquisition (supporting), investigation (supporting), methodology (supporting), visualization (supporting), writing – review and editing (supporting).

## Conflicts of Interest

The authors declare no conflicts of interest.

## Supporting information


**Appendix S1:** ece372198‐sup‐0001‐AppendixS1.docx.

## Data Availability

The data underlying this article is available in Zenodo, https://doi.org/10.5281/zenodo.15861625.
