# Supplementary material for: Decoupling of Radial Growth Phenology From Temperature Constraints in the Clonal Shrub Alnus alnobetula at the Alpine Treeline
Source: Ecol Evol. 2025 Sep 29;15(10):e72198. doi: 10.1002/ece3.72198 (PMC12479110; doi:10.1002/ece3.72198)
Supplement: Supplementary file 1 — Appendix S1: ece372198‐sup‐0001‐AppendixS1.docx. [file ECE3-15-e72198-s001.zip › ece372198-sup-0001-AppendixS1.docx]

**Appendix**

**Supplementary** **tables**

**Table S1.** Monthly mean daily air temperature and precipitation sum during May through September 2022–2024 recorded on top of Mt. Patscherkofel. Mean values±standard deviations are shown (doy = day of the year, Summer = June through August).

|  |  | Air temperature (°C) | | | Precipitation (mm) | | |
| --- | --- | --- | --- | --- | --- | --- | --- |
| Month | doy | 2022 | 2023 | 2024 | 2022 | 2023 | 2024 |
| May | 121-151 | 5.3 ± 4.1 | 2.9 ± 3.2 | 3.5 ± 2.0 | 72 | 164 | 50 |
| June | 152-181 | 9.8 ± 3.3 | 8.5 ± 3.0 | 7.2 ± 4.5 | 90 | 40 | 186 |
| July | 182-212 | 10.5 ± 3.2 | 9.9 ± 3.3 | 10.7 ± 2.9 | 123 | 165 | 106 |
| Aug | 213-243 | 10.0 ± 2.2 | 10.0 ± 5.3 | 12.5 ± 2.3 | 72 | 228 | 83 |
| Sep | 244-273 | 4.0 ± 4.6 | 9.8 ± 3.8 | 4.9 ± 5.4 | 85 | 68 | 128 |
| Summer | 152-243 | 10.1 ± 2.9 | 9.5 ± 4.0 | 10.2 ± 3.9 | 285 | 433 | 375 |

**Table S2.** Time of bud burst (BB) and delay between bud burst and onset of radial stem growth (ΔRG; see **Table 2**) in *Alnus alnobetula* at study plots located within the treeline ecotone during 2022–2024 (doy=day of the year, FL=forestline, TR-N=treeline north, TR-S=treeline south-east, M±SD =Mean±standard deviation). Different letters indicate statistically significant differences between ΔRG (Student’s *t*-test; *P*<0.01).

| Plot | 2022 | | 2023 | | 2024 | |  |
| --- | --- | --- | --- | --- | --- | --- | --- |
|  | BB (doy) | ΔRG (days) | BB (doy) | ΔRG (days) | BB (doy) | ΔRG (days) | ΔRG (days) M±SD |
| FL | 136 | 28 | 151 | 27 | 150 | 30 | 28±2^ab^ |
| TR-S | 138 | 33 | 148 | 28 | 150 | 32 | 31±3^b^ |
| TR-N | 146 | 26 | 157 | 26 | 161 | 24 | 25±1^a^ |

**Table S3.** Daily mean air temperature (T_air_, °C) and maximum daily mean T_air_ (T_air-mx_, °C) at and over a period of 7 and 14 days (7 d and 14 d, respectively) around the mean inflection point (IP) across the treeline ecotone (see **Table 3**) during study years 2022–2024 recorded on top of Mt. Patscherkofel. Mean values±standard deviations are shown (doy=day of the year).

|  | doy | T_air_ (°C)  2022 | doy | T_air_ (°C)  2023 | doy | T_air_ (°C)  2024 |
| --- | --- | --- | --- | --- | --- | --- |
| IP | 185 | 10.1 | 192 | 16.7 | 200 | 12.7 |
| 7 d | 182–188 | 9.5±2.6 | 189-195 | 12.7±3.1 | 197-203 | 12.0±1.5 |
| 14 d | 179–192 | 8.7±2.7 | 186-199 | 12.1±3.1 | 194-207 | 10.9±1.7 |
| T_air-mx_ | 201 | 16.8 | 236 | 17.4 | 224 | 16.8 |
| 7 d | 200–206 | 14.2±1.9 | 231-237 | 16.1±1.1 | 223-229 | 15.0±1.1 |
| 14 d | 194–207 | 13.0±2.1 | 224-237 | 14.9±1.6 | 217-230 | 13.5±2.0 |

**Table S4**. Spearman correlation coefficients (*ρ*) between environmental variables (daily means with the exception of precipitation, where daily sums were used) and daily radial increments of *Alnus alnobetula* during study years 2022–2024. Correlations were calculated over the period between 5 % and 95 % of the Gompertz-modelled growth, and considering of a lag of one day. The corresponding coefficients are shown after the back slash. Number of data points is 137–181 for FL (except n=74–77 for Prec), 144–182 for TR-S (except n=76–80 for Prec), and 113–136 for TR-N (except n=59–63 for Prec). FL=forestline; TR-N=treeline north; TR-S=treeline south-east; T_air_=air temperature, T_soil_=soil temperature, T_camb_=cambium temperature, RH=relative air humidity, VPD=daily mean vapour pressure deficit, Prec=precipitation, SWC=soil water content. ***=*P*<0.001; **=*P*<0.01; *=*P*<0.05

|  | T_air_ (°C) | T_soil_ (°C) | T_camb_ (°C) | RH (%) | VPD (kPa) | Prec (mm) | SWC (%) |
| --- | --- | --- | --- | --- | --- | --- | --- |
| FL | -0.026\0.272*** | 0.064\0.053 | 0.103\0.359*** | 0.178*\-0.280*** | -0.159*\0.285*** | 0.207\-0.160 | 0.126\0.092 |
| TR-S | 0.250***\0.456*** | 0.258***\0.200** | 0.205*\0.424*** | 0.024\-0.417*** | 0.063\0.446*** | 0.322**\-0.059 | 0.038\-0.065 |
| TR-N | 0.111\0.307*** | 0.263**\0.260** | 0.177\0.371*** | 0.137\-0.134 | 0.089\0.381*** | 0.066\-0.140 | -0.027\-0.122 |
