# Supplementary material for: Decoupling of Radial Growth Phenology From Temperature Constraints in the Clonal Shrub Alnus alnobetula at the Alpine Treeline
Source: Ecol Evol. 2025 Sep 29;15(10):e72198. doi: 10.1002/ece3.72198 (PMC12479110; doi:10.1002/ece3.72198)
Supplement: Supplementary file 1 — Appendix S1: ece372198‐sup‐0001‐AppendixS1.docx. [file ECE3-15-e72198-s001.zip › TableS1.pdf]

Table S1. Monthly mean daily air temperature and precipitation sum during May through September 2022–2024 recorded on top of Mt. Patscherkofel. Mean values±standard deviations are shown (doy = day of the year, Summer = June through August).

		Air temperature (°C)			Precipitation (mm)		
Month	doy	2022	2023	2024	2022	2023	2024
May	121-151	5.3 ± 4.1	2.9 ± 3.2	3.5 ± 2.0	72	164	50
June	152-181	9.8 ± 3.3	8.5 ± 3.0	7.2 ± 4.5	90	40	186
July	182-212	10.5 ± 3.2	9.9 ± 3.3	10.7 ± 2.9	123	165	106
Aug	213-243	10.0 ± 2.2	10.0 ± 5.3	12.5 ± 2.3	72	228	83
Sep	244-273	4.0 ± 4.6	9.8 ± 3.8	4.9 ± 5.4	85	68	128
Summer	152-243	10.1 ± 2.9	9.5 ± 4.0	10.2 ± 3.9	285	433	375
