# Supplementary material for: Decoupling of Radial Growth Phenology From Temperature Constraints in the Clonal Shrub Alnus alnobetula at the Alpine Treeline
Source: Ecol Evol. 2025 Sep 29;15(10):e72198. doi: 10.1002/ece3.72198 (PMC12479110; doi:10.1002/ece3.72198)
Supplement: Supplementary file 1 — Appendix S1: ece372198‐sup‐0001‐AppendixS1.docx. [file ECE3-15-e72198-s001.zip › TableS2.pdf]

Table S2. Time of bud burst (BB) and delay between bud burst and onset of radial stem growth (ΔRG; see **Table 2**) in *Alnus alnobetula* at study plots located within the treeline ecotone during 2022–2024 (doy=day of the year, FL=forestline, TR-N=treeline north, TR-S=treeline south-east, M±SD =Mean±standard deviation). Different letters indicate statistically significant differences between ΔRG (Student's *t*-test; $P<0.01$).

Plot	2022		2023		2024		
	BB (doy)	ΔRG (days)	BB (doy)	ΔRG (days)	BB (doy)	ΔRG (days)	ΔRG (days) M±SD
FL	136	28	151	27	150	30	28 ± 2^{ab}
TR-S	138	33	148	28	150	32	31 ± 3^{b}
TR-N	146	26	157	26	161	24	25 ± 1^{a}
