# Supplementary material for: Decoupling of Radial Growth Phenology From Temperature Constraints in the Clonal Shrub Alnus alnobetula at the Alpine Treeline
Source: Ecol Evol. 2025 Sep 29;15(10):e72198. doi: 10.1002/ece3.72198 (PMC12479110; doi:10.1002/ece3.72198)
Supplement: Supplementary file 1 — Appendix S1: ece372198‐sup‐0001‐AppendixS1.docx. [file ECE3-15-e72198-s001.zip › TableS3.pdf]

Table S3. Daily mean air temperature (T_{air}, °C) and maximum daily mean T_{air} ($T_{air\text{-}mx}$, °C) at and over a period of 7 and 14 days (7 d and 14 d, respectively) around the mean inflection point (IP) across the treeline ecotone (see **Table 3**) during study years 2022–2024 recorded on top of Mt. Patscherkofel. Mean values±standard deviations are shown (doy=day of the year).

	doy	T_{air} (°C)	doy	T_{air} (°C)	doy	T_{air} (°C)
		2022		2023		2024
IP	185	10.1	192	16.7	200	12.7
7 d	182–188	9.5±2.6	189-195	12.7±3.1	197-203	12.0±1.5
14 d	179–192	8.7±2.7	186-199	12.1±3.1	194-207	10.9±1.7
$T_{air\text{-}mx}$	201	16.8	236	17.4	224	16.8
7 d	200–206	14.2±1.9	231-237	16.1±1.1	223-229	15.0±1.1
14 d	194–207	13.0±2.1	224-237	14.9±1.6	217-230	13.5±2.0
