# Supplementary material for: Decoupling of Radial Growth Phenology From Temperature Constraints in the Clonal Shrub Alnus alnobetula at the Alpine Treeline
Source: Ecol Evol. 2025 Sep 29;15(10):e72198. doi: 10.1002/ece3.72198 (PMC12479110; doi:10.1002/ece3.72198)
Supplement: Supplementary file 1 — Appendix S1: ece372198‐sup‐0001‐AppendixS1.docx. [file ECE3-15-e72198-s001.zip › TableS4.pdf]

Table S4. Spearman correlation coefficients (*ρ*) between environmental variables (daily means with the exception of precipitation, where daily sums were used) and daily radial increments of *Alnus alnobetula* during study years 2022–2024. Correlations were calculated over the period between 5 % and 95 % of the Gompertz-modelled growth, and considering of a lag of one day. The corresponding coefficients are shown after the back slash. Number of data points is 137–181 for FL (except n=74–77 for Prec), 144–182 for TR-S (except n=76–80 for Prec), and 113–136 for TR-N (except n=59–63 for Prec). FL=forestline; TR-N=treeline north; TR-S=treeline south-east; T_{air}=air temperature, T_{soil}=soil temperature, T_{camb}=cambium temperature, RH=relative air humidity, VPD=daily mean vapour pressure deficit, Prec=precipitation, SWC=soil water content. ***=P<0.001; **=P<0.01; *=P<0.05

	T_{air} (°C)	T_{soil} (°C)	T_{camb} (°C)	RH (%)	VPD (kPa)	Prec (mm)	SWC (%)
FL	-0.026\0.272***	0.064\0.053	0.103\0.359***	0.178*\-0.280***	-0.159*\0.285***	0.207\-0.160	0.126\0.092
TR-S	0.250***\0.456***	0.258***\0.200**	0.205*\0.424***	0.024\-0.417***	0.063\0.446***	0.322**\-0.059	0.038\-0.065
TR-N	0.111\0.307***	0.263**\0.260**	0.177\0.371***	0.137\-0.134	0.089\0.381***	0.066\-0.140	-0.027\-0.122
